# The Origin of Novel Avian Influenza A (H7N9) and Mutation Dynamics for Its Human-To-Human Transmissible Capacity

**DOI:** 10.1371/journal.pone.0093094

**Published:** 2014-03-26

**Authors:** Jin Peng, Hao Yang, Hua Jiang, Yi-xiao Lin, Charles Damien Lu, Ya-wei Xu, Jun Zeng

**Affiliations:** 1 Department of Computational Mathematics and Bio-Statistics, Metabolomics and Multidisciplinary Laboratory for Trauma Research, Institute for Emergency and Disaster Medicine, Sichuan Provincial People’s Hospital, Sichuan Academy of Medical Sciences, Chengdu, Sichuan Province, China; 2 Emergency Medicine Center, Sichuan Provincial People’s Hospital, Sichuan Academy of Medical Sciences, Chengdu, Sichuan Province, China; Deakin University, Australia

## Abstract

In February 2013, H7N9 (A/H7N9/2013_China), a novel avian influenza virus, broke out in eastern China and caused human death. It is a global priority to discover its origin and the point in time at which it will become transmittable between humans. We present here an interdisciplinary method to track the origin of H7N9 virus in China and to establish an evolutionary dynamics model for its human-to-human transmission via mutations. After comparing influenza viruses from China since 1983, we established an A/H7N9/2013_China virus evolutionary phylogenetic tree and found that the human instances of virus infection were of avian origin and clustered into an independent line. Comparing hemagglutinin (HA) and neuraminidase (NA) gene sequences of A/H7N9/2013_China viruses with all human-to-human, avian, and swine influenza viruses in China in the past 30 years, we found that A/H7N9/2013_China viruses originated from Baer’s Pochard H7N1 virus of Hu Nan Province 2010 (HA gene, EPI: 370846, similarity with H7N9 is 95.5%) and duck influenza viruses of Nanchang city 2000 (NA gene, EPI: 387555, similarity with H7N9 is 97%) through genetic re-assortment. HA and NA gene sequence comparison indicated that A/H7N9/2013_China virus was not similar to human-to-human transmittable influenza viruses. To simulate the evolution dynamics required for human-to-human transmission mutations of H7N9 virus, we employed the Markov model. The result of this calculation indicated that the virus would acquire properties for human-to-human transmission in 11.3 years (95% confidence interval (CI): 11.2–11.3, HA gene).

## Introduction

In February 2013, the first H7N9 influenza patient was found in eastern China, followed by multiple cases in March and April [1], [2]. By May 13, 2013, China’s health authorities reported 130 human cases, 35 of whom had died [2]. According to a report by the authorities, all these cases were infected with H7N9 virus (A/H7N9/2013_China) via avian sources [2]. So far, there has been no human-to-human transmission case. However, if human-to-human transmission ever occurred, an outbreak of influenza pandemic is quite possible. Because of the high fatality rate (mortality rate ∼27%), prevention of this pandemic is a global priority. Discovering the origin of H7N9 and predicting when it will become transmittable between humans is crucial for monitoring and prevention in China and other countries worldwide.

There are two important issues to be addressed in the very first place when studying the outbreak of new bird flu: 1) where does the virus originate? And; 2) whether the virus will develop the capacity for human-to-human transmission, and if yes, when?

To address the first issue, we have a set of well-established methods. We compared genetic distances between viral sequences and traced H7N9 back to its origin. However, with the recent progress in sequencing technology, the use of influenza genome sequencing data is growing quickly. This poses a new challenge: how to decide the appropriate strategy to screen for the candidate gene to obtain sequence alignment and establish the correct evolutionary phylogenetic tree?

Addressing the second issue is more difficult. Because of the high possibility of re-assortment and mutation, evolution of RNA viruses like influenza is quite different from other species [3]. Previous studies have tried to address the human-to-human transmission issue by studying quasi-evolution [4], [5]. Although some researchers suggested that the error catastrophe would lead to a biological gap between different strains of virus [6], [7], some researchers found that the virus can be “hidden” in the genome of other species and is able to cross the error catastrophe gap [8], [9]. In this case, influenza viruses could transfer from one strain to another through accumulation of mutations. Accordingly, we could compute the time required for the transformation from non-human-to-human virus to human-to-human transmissible virus from the mutation ratio.

We present here an interdisciplinary method to track the origin of A/H7N9/2013_China influenza virus and establish an evolutionary dynamics model for its mutation to a human-to-human transmittable variant.

## Results

The main process of evolutionary phylogenetic analysis is to calculate the distance between two sequences. Distance estimation is based on the expected number of nucleotide substitutions per site in a nucleic acid sequence. Continuous-time Markov model are commonly used for this purpose because the nucleotide sites in the sequence are generally assumed to evolve independently of each other [10]. The Markov model poses a property that has no memory. It means that when one nucleotide mutates into another, it only depends on its current state, not on how the current state is reached. We chose the JC69 model (Jukes and Cantor 1969) that assumes that every nucleotide has the same mutation rate (λ) [25].

**Figure 1:**
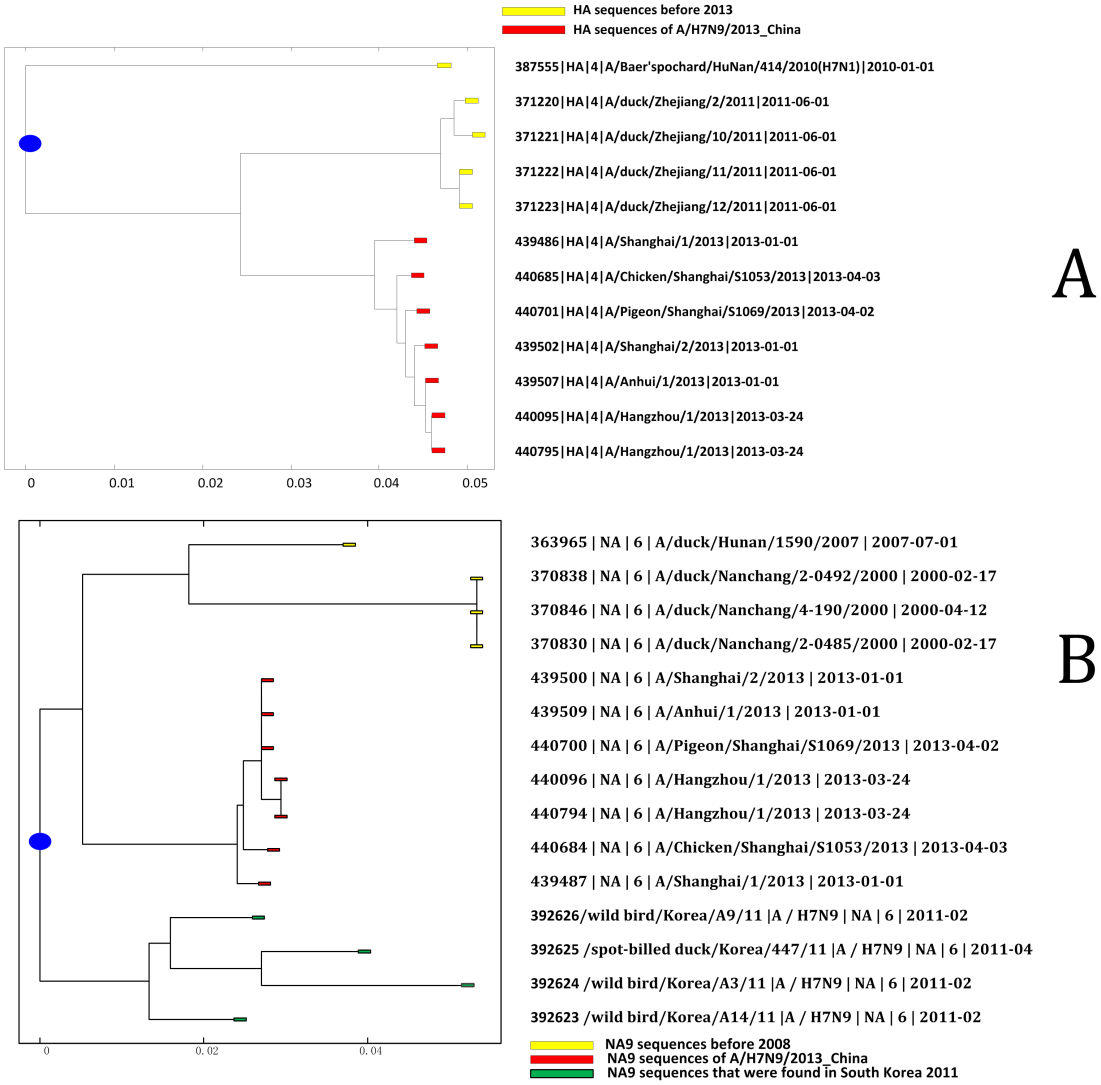
Phylogenetic tree of HA gene (A) and NA gene (B) of A/H7N9/2013_China and its Origin. The phylogenetic tree was generated by means of the JC69 distance-based method and using MatLab Bioinformatics Toolbox. (**A**) The A/H7N9/2013_China influenza viruses are in red and are clustered at the bottom of the tree. The yellow virus strain at the top of the tree is the original source of the HA gene of A/H7N9/2013_China – a 2010 H7N1 virus from Baer’s Pochard of Hu Nan. Some H7N1 viruses also shared high HA similarity with A/H7N9/2013_China from ducks of Zhe Jiang in 2011. A pigeon H7N9 virus from Shanghai also shared high similarity with A/H7N9/2013_China and is clustered with them near the bottom part of the tree. (**B**): The A/duck/Nanchang/2000/H2N9 and A/duck/Hunan/2007/H6N9 are in yellow and clustered at top of the tree. The A/H7N9/2013_China influenza viruses are in red and clustered at the middle part of the tree. Four South Korea avian H7N9 viruses from 2011 are in green and clustered at the bottom. The distance analysis indicated that the NA gene of viruses from Nanchang and Hu Nan are closer to A/H7N9/2013_China than to the viruses from South Korea.

After analyzing phylogenetic relations between A/H7N9/2013_China and other influenza viruses from China in the past 30 years, we found 95.5% similarity in the hemagglutinin (HA) genes between A/H7N9/2013_China and an H7N1 avian influenza virus of a water bird (A/Baer’s Pochard/Hunan/2010/H7N1, EPI 387555) from Hu Nan Province (Figure 1A). Similarity of neuraminidase (NA) genes between A/2013/H7N9_China and the H2N9 virus obtained from ducks in Nan Chang, a city in Jiang Xi Province of China (A/duck/Nanchang/2000/H2N9) was 96.7% (EPI370846, 370830, and 370838) (Figure 1B). In addition, we found an H6N9 avian influenza virus in ducks from Hu Nan Province of China (A/duck/Hunan/2007/H6N9, EPI363965) in 2007, which shared 94.3% similarity with the NA gene with A/H7N9/2013 (Figure 1B). We also found H7N3 viruses in ducks from Zhe Jiang Province of China (A/duck/H7N3/2011/Zhejiang, EPI 371220) in 2011 shared 95.9% and 95.7% (EPI371221, 371222, and 371223) similarities with the HA gene of A/H7N9/2013_China (Figure 1A), respectively.

**Figure 2:**
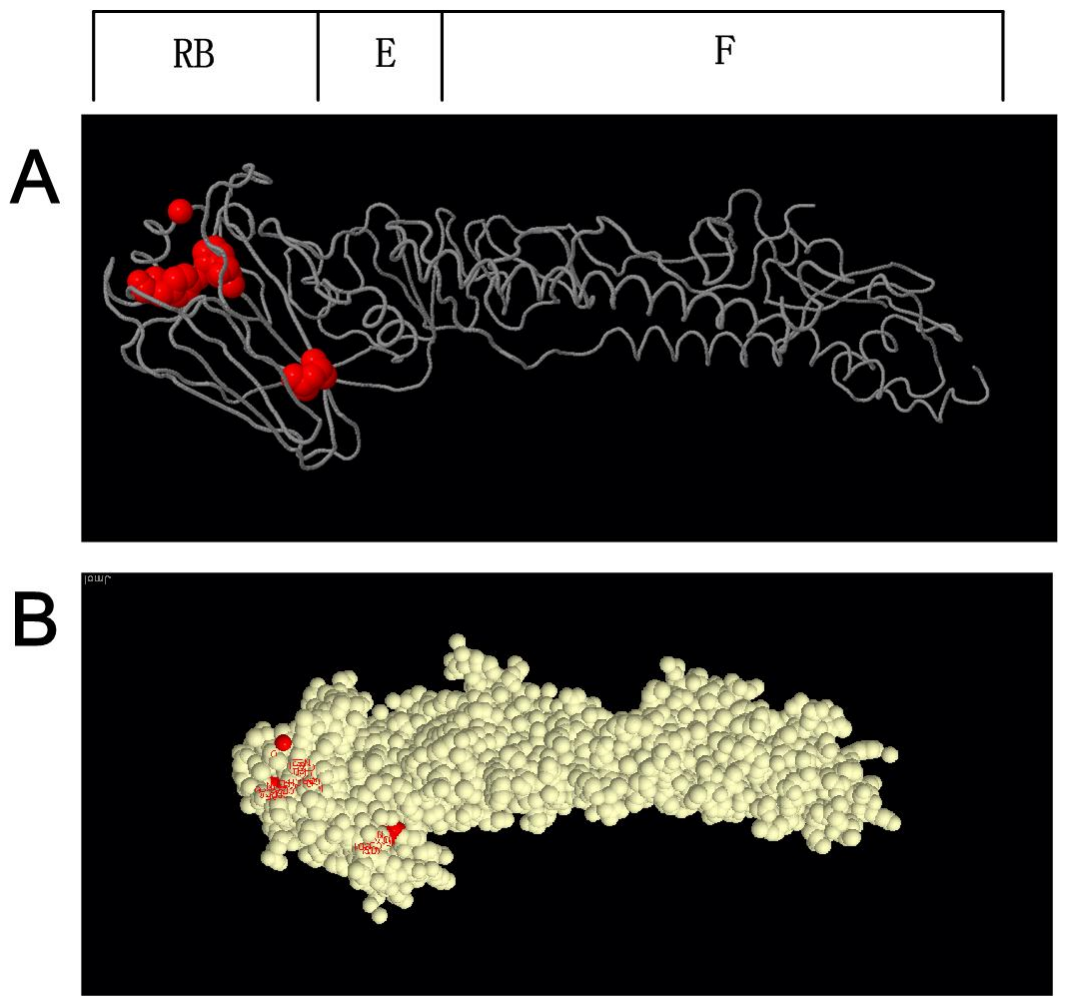
Tertiary structures of HA protein of A/H7N9/2013_China (A). Trace diagram of HA monomer, loci S183D V195G L235Q I335T N410T D455N and V541A are mutated loci and are marked by red space-filled atoms. Mutated loci are all located at the receptor-binding (RB) domain. (**B**): **The space-filled atom diagram of HA protein.** Red spots are mutated loci that are inside of the head of the protein. There is no change in the fusogenic stalk (**F**) and the esterase (**E**) domain.

Comparing amino acid sequences of HA of A/H7N9/2013_China and A/duck/H7N3/2011/Zhejiang (EPI 371220), we found that mutations occurred at S183D, V195G, L235Q, I335T, N410T, D455N, and V541A. Tertiary structural analysis found that these loci are in close spatial proximity (Table 1, Figure 2).

**Table 1:** Mutated loci of HA and NA proteins.

Virus	Accessionno. (HA)	Accessionno. (NA)	Mutated Loci of HA protein	Mutated Loci of NA protein
A/Shanghai/2/2013 H7N9	439502	439500	S183D V195G L235Q I335T N410TD455N V541A	70–74 deletion T83A T86NA403T G42S
A/Anhui/1/2013 N7N9	439507	439509	Same as above	Same as above
A/Hangzhou/1/2013 H7N9	440095	440096	Same as above	Same as above
A/Hangzhou/1/2013	440795	440794	Same as above	Same as above
A/Pigeon/Shanghai/S1069/2013 H7N9	440701	440700	Same as above and I253V	Same as above
A/Chicken/Shanghai/S1053/2013 H7N9	440685	440684	Same as above	Same as above, andR40K,V59I,V307I,I352V
A/Shanghai/1/2013 H7N9	439486	439487	Same as A/Shanghai/2/2013 H7N9,and S146A, N183S,T230P,Y292H	Same as A/Shanghai/2/2013 H7N9
A/duck/Zhejiang/2/2011 H7N3	371220	N/A	K128E,R181K,V188I,V211	N/A
Baer’s Pochard/Hunan/414/2010(H7N1)	387555	N/A	I11V,I56V,I95V,E122K,T130A,Y167N,L176S,N191I,T197A,S208N	N/A
A/duck/Hunan/1590/2007 (H6N9)	N/A	363965	N/A	V18I,T19I,A275T,S401L,G418E,D461N,V361A
A/duck/Nanchang/4-190/2000 |(H2N9)	N/A	370846	N/A	N116S,K176R,V361A
A/wild bird/Korea/A9/11 A/H7N9	N/A	392626	N/A	P24A,I24V,T29A,I119V

N/A: not applicable.

Next, we considered the time required for A/H7N9/2013 to become a human-to-human transmittable virus. It is well known that HA is a key gene for human-to-human transmission [11]. We found 38 mutations in the HA7 amino acid sequence of influenza viruses since 2010. We then employed the Markov model to compute the mutation velocity of this sequence of HA7 amino acids and established a mutation dynamics model. We determined that A/H7N9/2013_China could become human-to-human transmittable in 11.3 years (95% CI: 11.2–11.3) by mutations in the HA gene.

## Discussion

The evolution of avian influenza virus is quite different from that of eukaryotic species in that it is a kind of quasi-species evolution. In this situation, high mutation rates and accumulation of errors will cause error catastrophe and lead to extinction. However, genetic re-assortment provides a shelter mechanism to preserve certain gene sequences. In other words, a harmful gene could be conserved within a genetic pool. In terms of the origin of A/H7N9/2013_China, we found that crucial sequences of this virus have existed in water birds for a long time.

**Figure 3:**
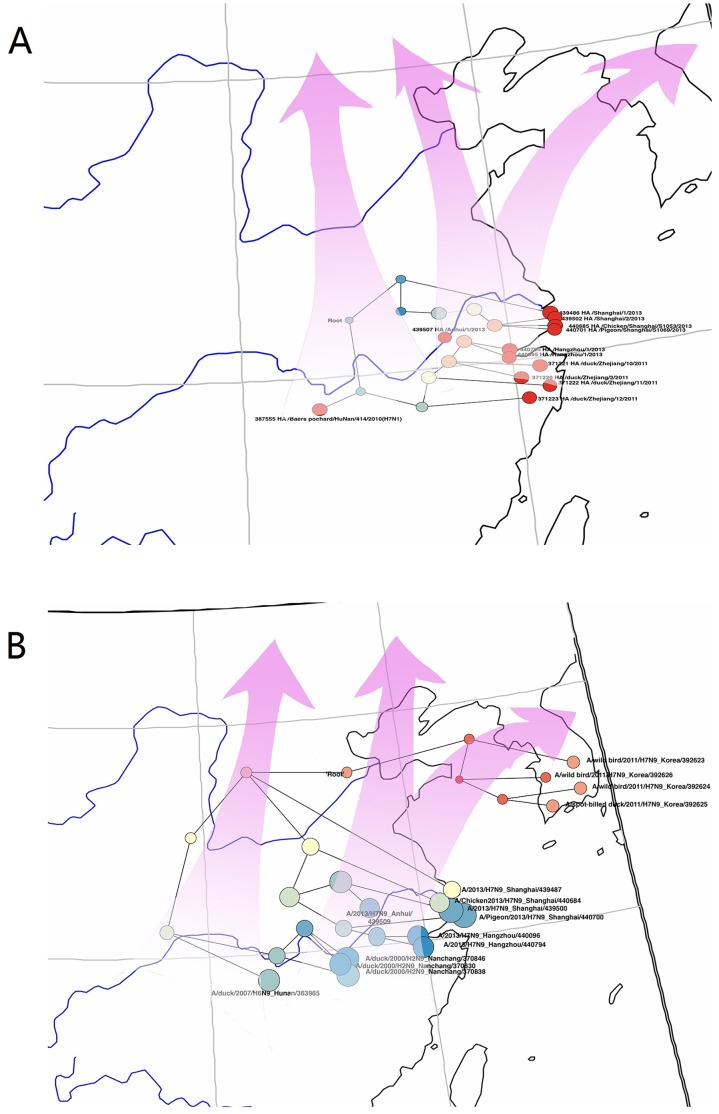
Spatial distribution of closest HA (A) and NA genes (B) of H7N9 in China. Red arrow indicates direction of migration of water bird.

Nanchang is a city in Jiang Xi Province and is close to Po Yang Lake, the largest body of water in China. The second largest body of water in China, Dong Ting Lake, is in Hu Nan Province. The two lakes are linked by the Yangzi River. Every winter, migrant water birds fly from northwestern to southern China; Po Yang and Dong Ting lakes are important winter habitats for these birds and the wet land of Zhe Jiang Province is a major water stop on the birds’ migration route (Figure 3A, 3B). The HA gene of A/H7N9/2013 most likely came from birds in Dong Ting Lake. The NA gene of A/H7N9/2013 most likely came from birds in Po Yang Lake and persisted in the Po Yang-Dong Ting water system. In southern and eastern China, ducks generally are bred in a natural water body and could easily contract influenza viruses from wild birds [8], [23]. We therefore conclude that A/H7N9/2013 is a re-assortment virus that acquired HA and NA genes from migrant water birds in China.

In April 2013, Chinese Center for Disease Control and Prevention (China CDC) published an article on the novel H7N9 viruses [12]. In their opinion, the origin of A/H7N9/2013 derives from the re-assortment of the HA gene of A/duck/Zhejiang/12/2011(H7N3) and the NA gene of A/wild_bird/Korea/A14/2011(H7N9)_South_Korea. We do not agree with China CDC on their explanation of the origin of A/H7N9/2013. We argue this because China CDC’s research did not include A/Baer’s Pochard/Hunan/2010/H7N1, A/duck/Nanchang/2000/H2N9 and A/duck/Hunan/2007/H6N9 in their phylogenetic tree; although we found that A/duck/Zhejiang/12/2011 (H7N3) shared 95.5% similarity with A/H7N9/2013, which is consistent with China CDC’s findings. All three highly homological influenza viruses existed before A/duck/Zhejiang/12/2011(H7N3) and wild bird influenza viruses from South Korea (H7N9). Our phylogenetic analysis indicates that the South Korea wild bird influenza virus shares high similarity with A/H7N9/2013_China (96.3%), and it shares 95.7% similarity with A/duck/Nanchang/2000/H2N9. This indicates that A/duck/Nanchang/2000/H2N9 is a common ancestor of the NA genes of both A/H7N9/2013 and A/wild_bird/Korea/A14/2011(H7N9) South_Korea (Figure 1B). In addition, if wild birds migrated through South Korea, they would enter through northeastern China and not through Zhejiang, Anhui, and Shanghai [13]–[17] (Figure 3A and B).

It is very common to explore the source of a virus by sequence alignment. However, with increasingly massive amounts of the virus genome data becoming available, it is not realistic to compare all the flu virus strains by sequence alignment. Therefore, it is necessary to find a new strategy to identify the origin of the H7N9 virus. Unlike the study by Liu et al [22], we believe the HA and NA genes restructured into other virus strains and discarded the other genes that have no direct relationship with the recognition between the epithelium mucosa and virus capsid. Through the above research, we found the HA7 of A/H7N9/2013_China originated from water birds from the Yangzi River system and existed in this region for at least three years. It did not originate from South Korea. Liu et al tried to identify the origin of A/H7N9/2013_China using a very different strategy of comparing sequences of all H7N9 viruses [22]. Considering the fast re-assortment and quasi-evolution of avian influenza and the migration of water birds, Liu’s strategy is not appropriate. Furthermore, we found NA9 sequences from Hu Nan (H6N9), Nan Chang (H2N9) and a HA7 sequence from Hu Nan (H7N1) exhibited high similarity with A/H7N9/2013_China, and the similarity between these sequences in China is significantly higher than that between A/H7N9/2013_China and H7N9 from South Korea. Indeed, a recent study by Zhu et al [23] found that the viruses from southern China’s water birds are the major contributors of the 2013 H7N9 influenza epidemic, and this is consistent with our result.

Quasi-evolution explains why a virus can transfer from one strain to another. Generally speaking, quasi-evolution is the accumulation of a replicating mutant spectrum with interfering mutant genomes prompted by enhanced mutagenesis. This process plays a key role in the sharp transition of virus populations into error catastrophe that leads to virus extinction. Nevertheless, when we consider the impacts of both re-assortment and quasi-evolution, we found that extinction and the diminishment of specific genes are two different things. HA7 and NA9 genes could drift between different avian virus quasi-species. The original virus H6N9_Hunan _1997 and H2N9_Nanchang_2001 may have been extinct in birds, but their sequences are still preserved and transmitted among the bird populations.

Although the mutated loci of HA protein of A/H7N9/2013_China in its primary structures are far apart from each other, all mutated loci in tertiary structures are close by. For HA protein, all mutated loci are located in spherical heads, which contain the sialic acid binding sites. This is a clue to why H7N9 avian influenza viruses posed limited poultry-to-human transmittable capacity.

We examined the time required for A/H7N9/2013 to become a human-to-human transmittable virus. We determined it could have the capacity of human-to-human transmission in 11.3 years [95% CI: 11.2–11.3 years, HA gene]. As we know, the interval between the influenza pandemics of 1957/1958 and 1968/1969 was around 10 years [18], [19], and these two pandemics originated from Hong Kong, China. Therefore we concluded that if the next pandemic originates from the current bird-to-human A/H7N9/2013_China by means of mutation, it could quite possibly happen in 2023/2024. Nevertheless, we should be cautious, as this prediction is fairly conservative because re-assortment is not considered in the current model.

According to our model, the duration of this process is approximately 11 years. This has been observed in previous studies [20], [21]. However, this phenomenon was not fully explained before our work. Some researchers have noted that the solar cycle may be the cause of bird flu cyclical outbreaks [20], [21], but our research suggests that it is likely that the virus mutation rate is the root cause.

Up to now, it has been impossible to predict outbreaks of influenza epidemics. To the best of our knowledge, our study is the first to provide a mathematical framework to understand the outbreak of influenza and to determine specific gene mutation sequences of avian flu virus required for human-to-human transmission capacity by mutation, and this process could be simulated by continuous-time Markov model.

## Materials and Methods

Our work included five steps: 1) clustering HA and NA genes of A/H7N9/2013_China and all influenza viruses (HA7 or NA9) from human, avian and swine of China since 1983 into the phylogenetic tree; 2) measuring distances between A/H7N9/2013_China and the viruses from the nearest nodes of the phylogenetic tree and finding ancestors of H7N9; 3) comparing amino acid sequences of HA protein between A/H7N9/2013_China and closely related viruses to screen mutated loci; 4) locating mutated loci in the tertiary structures of the HA protein; 5) employing the Markov model to establish evolutionary dynamics and to predict how long will it take for the avian A/H7N9/2013_China virus to mutate into a human-to-human transmittable virus.

We obtained gene sequences from A/H7N9/2013_China and all other influenza viruses from human, swine and avian in China in 1983–2013 from the Global Initiative on Sharing All Influenza Data (GISAID) website [24]. We transformed the original data to readable structures for MatLab (2011b, MathWorks Inc., Natick, MA, US) using E-Utilities software. These MatLab readable structures represented eight genes of the influenza virus. We used the Thompson-Higgins-Gibson algorithm to align gene sequences. The JC69 algorithm was used to establish a phylogenetic tree for all the viruses [10], [25].

Gene mutations caused amino acid mutations. In amino acid sequences of HA protein in influenza virus, mutations at key loci determines bird-to-human and/or human-to-human transmission capacity. Thus, we focused on amino acid sequence mutations of HA protein of A/H7N9/2013_China. Analyzing the phylogenetic relations of HA genes between A/H7N9/2013_China and other human-to-human transmittable influenza viruses, we found that the closest neighbors of H7N9 are two H3N2 viruses from Kunming (A/Kunming/H3N2/2005, EPI356039) and Nanjing (A/Nanjing/H3N2/1983, EPI92288) of China. We then set HA amino acid sequences and their tertiary structures of H3N2 influenza virus as comparison subjects. We obtained typical HA (ExPDB ID: 1ti8) amino acid sequences and their tertiary structures of H3N2 from Protein Data Bank (http://www.rcsb.org/pdb). We used the getpdp function from MatLab Bioinformatics Toolbox to re-construct the tertiary structures of A/H7N9/2013_China and visualized the mutated amino acid loci in protein HA.

Mutation velocity of A/H7N9/2013_China was calculated based on the comparison of HA amino acid sequence variations between viruses from seven H7N9 cases. The dates of illness onset were obtained from China CDC’s report. We set the closest human-to-human transmittable virus (H3N2) HA amino acid sequence as the outcome and entered A/H7N9/2013_China into the Markov model to calculation the time required for A/H7N9/2013_China to become human-to-human transmittable.

In summary, the mutation dynamics for A/H7N9/2013_China to become a human-to-human transmittable virus was calculated by the following process:

We used the JC69 algorithm [25], [26], which is the following continuous-time Markov transition probability matrix:
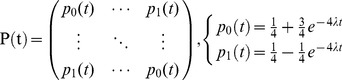
(1)


Then, we used the following formula to estimate mutation velocity.
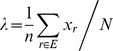
(2)Where, λ is the average amino acid residue mutation velocity, Xr is the number of mutated sites of r-th H7N9 HA/NA amino acids sequence; r is an index variable and N is the total number of amino acids residues.

The distance was estimated by
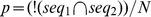
(3)




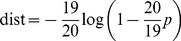
(4)


Then, 95% CI estimation is:
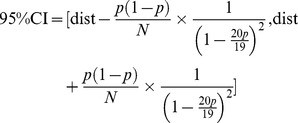
(5)Where p is the mutation ratio between the original A/H7N9/2013_China and the human-to-human H7N9 virus (mutated amino acid residues/total amino acid residues); dist is the distance between the two amino acid sequences.

Then we used the distance and mutation velocity to calculate the time when H7N9 becomes a human-to-human transmittable virus.
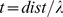
(6)

